# Mitochondrial DNA damage in non-melanoma skin cancer

**DOI:** 10.1038/sj.bjc.6600773

**Published:** 2003-01-28

**Authors:** S E Durham, K J Krishnan, J Betts, M A Birch-Machin

**Affiliations:** Department of Dermatology, School of Clinical and Laboratory Sciences, University of Newcastle, Leech Building, Medical School, Framlington Place, Newcastle upon Tyne NE2 4HH, UK

**Keywords:** skin cancer, MtDNA, deletion spectrum, tandem duplications, perilesional skin

## Abstract

Mitochondrial DNA (mtDNA) damage, predominantly encompassing point mutations, has been reported in a variety of cancers. Here we present in human skin, the first detailed study of the distribution of multiple forms of mtDNA damage in nonmelanoma skin cancer (NMSC) compared to histologically normal perilesional dermis and epidermis. We present the first entire spectrum of deletions found between different types of skin tumours and perilesional skin. In addition, we provide the first quantitative data for the incidence of the common deletion as well as the first report of specific tandem duplications in tumours from any tissue. Importantly, this work shows that there are clear differences in the distribution of deletions between the tumour and the histologically normal perilesional skin. Furthermore, DNA sequencing of four mutation ‘hotspot’ regions of the mitochondrial genome identified a previously unreported somatic heteroplasmic mutation in an SCC patient. In addition, 81 unreported and reported homoplasmic single base changes were identified in the other NMSC patients. Unlike the distribution of deletions and the heteroplasmic mutation, these homoplasmic mutations were present in both tumour and perilesional skin, which suggests that for some genetic studies the traditional use of histologically normal perilesional skin from NMSC patients may be limited. Currently, it is unclear whether mtDNA damage has a direct link to skin cancer or it may simply reflect an underlying nuclear DNA instability.

Each human cell contains hundreds to several thousand copies of the 16.5 kb human mitochondrial genome. This closed circular genome encodes 13 polypeptides of the respiratory chain complexes, as well as 22 transfer RNAs and two ribosomal RNAs used in mitochondrial protein synthesis. Compared to nuclear DNA, mitochondrial DNA (mtDNA) is highly susceptible to damage because it is not associated with protective histones, it is continually exposed to high levels of reactive oxygen species (ROS) generated by oxidative phosphorylation, and there is a limited capacity for mtDNA repair (Wallace, 1992). The complete mtDNA sequence was determined in 1981 (Anderson *et al*, 1981) and resequenced in 1999 (Andrews *et al*, 1999). A growing collection of reported mtDNA mutations and rearrangements has been associated with muscle and neurodegenerative diseases (Brierley *et al*, 1998; Chinnery *et al*, 1999).

Mitochondria have been implicated in the carcinogenic process because of their role in apoptosis (Kroemer and Reed, 2000) and other aspects of tumour biology; in particular, somatic mutations of mtDNA have been observed in a wide variety of human tumours (reviewed in Penta *et al*, 2001). These findings together with the fact that ultraviolet radiation (UVR) is important in the development of nonmelanoma skin cancer (NMSC), and has been shown to induce mtDNA damage in human skin (Birch-Machin *et al*, 1998; Ray *et al*, 2000), which led us to perform the first detailed study of mtDNA damage in human NMSC. This type of skin cancer consists of basal cell and squamous cell carcinoma (i.e. BCC and SCC, respectively). BCCs are the commonest form of skin cancer and occur mainly on sun-exposed body sites in elderly or middle-aged subjects. They arise from the basal keratinocytes of the epidermis, are locally invasive but rarely metastasise. SCCs are derived from moderately differentiated keratinocytes. They mainly occur in people over 55 years (y) of age and are found on sun-exposed sites. In contrast to BCCs, the SCCs are derived from precursor lesions such as actinic keratoses and importantly the SCCs may metastasise.

This study is the first of its kind in the skin cancer literature as it provides a detailed investigation of the different types of mtDNA damage in both SCCs and BCCs compared to matched histologically normal perilesional tissue. We have investigated the entire spectrum of large-scale deletions, the incidence of the common deletion and tandem duplications, and the distribution of single base changes in the mitochondrial genome.

## MATERIALS AND METHODS

### Patient samples and DNA extraction

Tumour and matched perilesional skin samples were taken with informed consent from patients undergoing excision of an NMSC, namely BCC (*n*=5, age range 55–89 y, mean 78 y) or an SCC (*n*=5, age range 70–87 y, mean 78 y) at the Out-Patients Clinic, Royal Victoria Infirmary, Newcastle, UK. The perilesional skin was split into dermis and epidermis, and DNA extracted as described previously (Birch-Machin *et al*, 1998).

### Long template PCR

The deletion spectrum of large-scale deletions was investigated by amplification of almost the entire 16.5 kb mitochondrial genome in two fragments, using long template PCR as described previously (Ray *et al*, 2000). To ensure reproducibility and to prevent any preferential amplification, the amount of DNA was standardised for each sample.

### 3-primer PCR

A competitive, radioactive, PCR assay was used to quantify the ratios of both deleted and wild-type mtDNA (Sciacco *et al*, 1994). Wild-type and deleted mtDNAs are demonstrated by the presence of a 755 and a 470 bp PCR product, respectively.

### ‘Back-to-Back’ primer assay

This methodology has been previously described to screen for the presence of tandem duplications in the D-loop (Brockington *et al*, 1993). The primers were L336 (nucleotide position (np) 336–355) and H335 (np 335–316). Under the designated PCR conditions, the orientation of these primers prevents the generation of a product from wild-type mtDNA but permits a product from mitochondrial genomes harbouring a 200 bp or a 260 bp tandem duplication in the D-loop. The nucleotide positions of the tandem duplication breakpoints have been previously described (Brockington *et al*, 1993; Lee *et al*, 1994).

### DNA sequence analysis

Direct DNA sequencing of ND1, ND5, 16 s RNA and the noncoding D-loop region of the mitochondrial genome was performed as previously described (Andrews *et al*, 1999). A review of the current literature shows that these four mtDNA regions appear to be highly susceptible to mutations (Penta *et al*, 2001). Automated DNA sequencing was performed by MWG Biotech (Ebersberg, Germany) and the resulting sequences compared to the revised Cambridge mtDNA reference sequence (MITOMAP). Confirmation of unreported base changes was performed by sequencing in the reverse direction on independent PCR products.

## RESULTS

### The spectrum of large-scale mtDNA deletions

**Figure 1:**
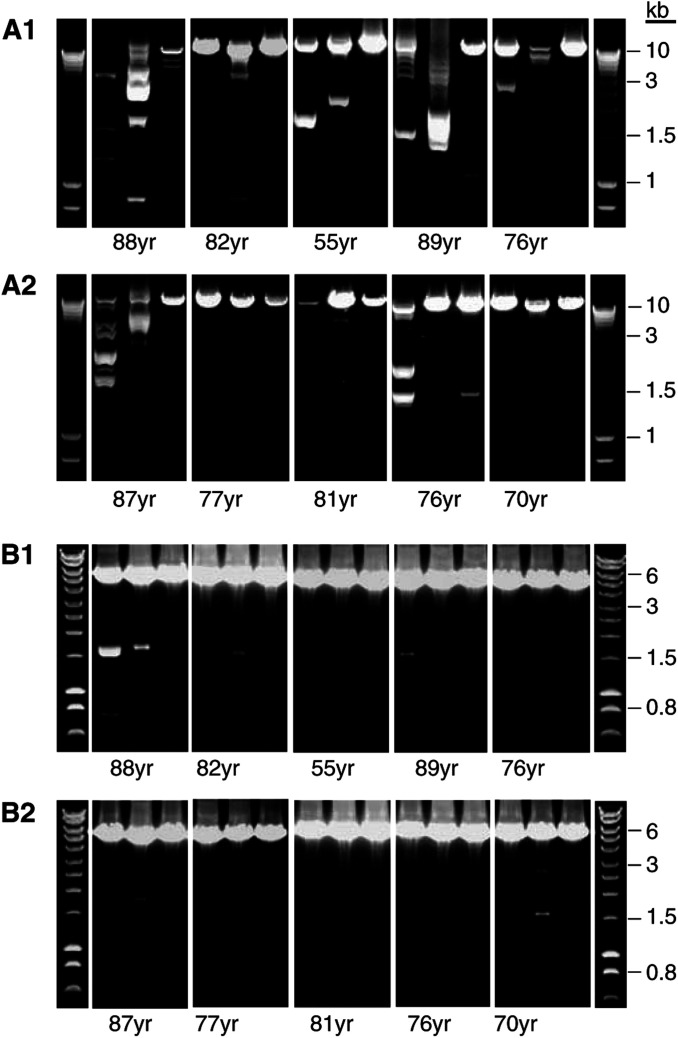
MtDNA deletion spectrum of tumour and perilesional skin. **A1** and **A2**: 11 kb PCR profile for BCC and SCC, respectively. **B1** and **B2**: 5 kb PCR profile for BCC and SCC, respectively. Each panel of three lanes represents a single NMSC patient; lane 1=tumour, lane 2=dermis, lane 3=epidermis. The observed deletion profiles were reproducible over three independent PCR experiments. Molecular weight markers (Hyperladder I-range 10 kb–200 bp, Bioline Ltd, London UK) are the single lanes at the ends of each row of panels.

The deletion spectrum was investigated in the mitochondrial genomes from the tumour samples and the histologically normal perilesional dermis and epidermis samples from each NMSC patient (total sample number is 30). Almost the entire mitochondrial genome was amplified in two fragments, namely 11 and 5.5 kb. The spectrum of deletions is visualised as a DNA ladder of PCR products on an agarose gel (Figure 1

). The 11 kb PCR fragment encompasses the major deletion arc where the vast majority of mtDNA deletions are reported (MITOMAP). There were 20 different-sized deletions observed in both the tumour and perilesional skin, with BCCs harbouring more deletions than SCCs (Figure 1, A1 and A2, respectively). Within each triplet of patient samples, the number of deletions in the epidermis never exceeded that observed in the corresponding tumour and dermis. Interestingly, the observed pattern of deletions in the tumour was often very different compared to that observed in the matched perilesional skin. Despite this, there is not a clear deletion pattern that is characteristic of a particular tumour type.

The 5.5 kb PCR fragment encompasses the minor deletion arc of the mitochondrial genome, where fewer deletions are reported (MITOMAP). A total of only four different-sized deletion species were observed but, like the 11 kb PCR results, the greatest number of total deletions was observed in the BCC as opposed to the SCC tumour samples (Figure 1, B1 and B2, respectively). Interestingly, a PCR fragment of approximately 1.5 kb (equivalent to a 4 kb deletion) was found to be common to all those samples that harboured any deletion(s).

### Quantification of the 4977 bp common mtDNA deletion

**Figure 2:**
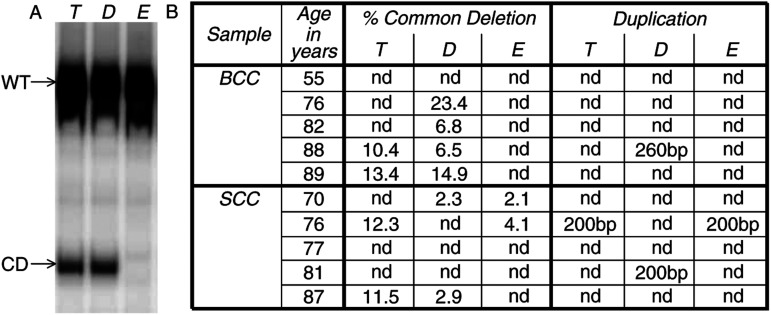
Quantification of the 4977 bp common deletion and detection of tandem duplications. (**A**) An example of a phosphorimage from the 3-primer PCR assay showing the PCR products that represent wild-type (WT) and deletion products (CD) in the tumour (T), and perilesional dermis and epidermis (D and E, respectively) from the 89-y-old BCC patient detailed in part **B**. (**B**) The triplet of samples representing tumour (T), perilesional dermis (D) and epidermis (E) from each NMSC patient were screened for the common deletion and duplications. nd=not detected (duplications); levels below 2% (common deletion).

Low levels of the common deletion, typically much less than 0.2%, have been associated with ageing (Nagley and Wei, 1998). To ensure that the effects of ageing do not confound our results for the common deletion, we have presented data representing only high levels (i.e. >2%) of deletion (a typical example is shown in Figure 2A

). We have shown previously that these high deletion levels are not associated with chronological ageing, and this is further confirmed by the data in Figure 2B that is arranged in ascending order of age. The high levels of the common deletion varied in the NMSC samples from 2.3 to 23.4% but were not observed in the perilesional epidermis from the BCC patients. Four of the 10 NMSC patients harbour high deletion levels in both the tumour and the corresponding perilesional skin. In three of these four patients, it was the tumour that harboured the highest level of deletion by a factor of approximately 2–4-fold. Even in the outlying sample (i.e. BCC, 89 y) the levels of deletion in the tumour and perilesional skin were similarly high at 13.4 and 14.9%, respectively. Interestingly, in a manner similar to the spectrum of deletions, the observed patterns of the common deletion in the tumour was often very different compared to that observed in the matched perilesional skin.

### Detection of tandem duplications within the D-loop

A total of four samples, representing three different NMSC patients, were found to harbour a 200 or 260 bp tandem duplication. We found one BCC patient with a 260 bp duplication and three SCC patients with a 200 bp duplication (Figure 2B). It has been proposed previously that there is a link between the presence of duplications and the common deletion (Nagley and Wei, 1998). In this respect, three of the four samples with a duplication also contained the common deletion in the corresponding sample.

### MtDNA base changes

Comparative DNA sequence analysis was performed on four regions of the mitochondrial genome from the tumour samples and the histologically normal perilesional dermis and epidermis samples from each NMSC patient. A total of 81 homoplasmic and one heteroplasmic base substitutions were identified (Table 1

**Table 1:** Summary of the 82 different mtDNA base changes identified in the tumour and perilesional skin samples. (**A**) Base changes that have been previously unreported in the MITOMAP database. (**B**) Previously reported changes that alter an amino acid. (**C**) Previously reported synchronous changes that do not alter the amino acid

**Base change**	**Map locus**	**Amino-acid change**	**Base change**	**Map locus**	**Amino-acid change**
(A)					
C438T^a^	OHR/LSP/TFL	Noncoding	C12348T	ND5	—
A574G	D-loop	Noncoding	C12633A	ND5	—
C494A	D-loop	Noncoding	C13251T	ND5	—
T1694C	RNR2	–	A13528G	ND5	Thr–Ala
A2294G	RNR2	–	C13565T	ND5	Ser–Phe
G3531A	ND1	–	A13614G	ND5	—
C4832T	ND2	–	A16183G	HV1/7SDNA	Noncoding
					
**Base change**	**Map locus**	**Amino-acid change**	**Base change**	**Map locus**	**Amino-acid change**
(B)					
T4216C	ND1	Tyr–His	G13708A	ND5	Ala–Thr
A13105G	ND5	Iso–Val	C13934T	ND5	Thr–Met
A13117G	ND5	Iso–Val			
					
**Base change**	**Map locus**	**Base change**	**Map locus**		
(C)					
A73G	HV2/7SDNA	A12308G	ND2		
C150T	HV2/OHR/7SDNA	G12372A	ND5		
T152C	HV2/OHR/7SDNA	A12612G	ND5		
G185A	HV2/OHR/7SDNA	C12813T	ND5		
T195C	HV2/OHR	G13368A	ND5		
G228A	HV2/OHR/CSB1	A14139G	ND5		
T239C	HV2/TFX/OHR	C14167T	ND5		
A263G	HV2/OHR	G15928A	TT		
C295T	HV2/TFY/OHR	C16069T	7SDNA		
n(C)303	HV2/CSB2/TFY/OHR	T16093C	7SDNA		
n(C)311	HV2/CSB2/OHR	T16126C	7SDNA		
C456T	D-loop	A16162G	HV1/TAS/7SDNA		
C462T	D-loop	A16163G	HV1/TAS/7SDNA		
T489C	D-loop	A16166G	HV1/TAS/7SDNA		
C497T	D-loop	n(C)16184	HV1/7SDNA		
G499A	D-loop	C16186T	HV1/7SDNA		
n(CA)514	D-loop	T16189C	HV1/7SDNA		
n(C)568	D-loop	T16209C	HV1/7SDNA		
G709A	RNR1	T16224C	HV1/7SDNA		
T1189C	RNR1	C16256T	HV1/7SDNA		
A1438G	RNR1	C16286T	HV1/7SDNA		
A1811G	RNR2	C16294T	HV1/7SDNA		
G1888A	RNR2	T16304C	HV1/7SDNA		
A2706G	RNR2	T16311C	HV1/7SDNA		
G3010A	RNR2	A16343G	HV1/7SDNA		
T3290C	TL1	T16356C	HV1/7SDNA		
A3480G	ND1	T16362C	HV1/7SDNA		
G3915A	ND1	G16390A	7SDNA		
T4646C	ND2	C16400T	7SDNA		
T4703C	ND2	A16482G	7SDNA		
A4727G	ND2	T16519C	7SDNA		
A4769G	ND2				

The abbreviations for the map loci are detailed in MITOMAP.

a All changes are homoplasmic and occur in both tumour and perilesional skin except this heteroplasmic somatic mutation.

). In those cases where a homoplasmic base substitution was detected in the tumour, the same DNA change was also observed in both the perilesional dermis and epidermis. This contrasts with the distribution of the deletions in these samples, as described in the previous section.

**Figure 3:**
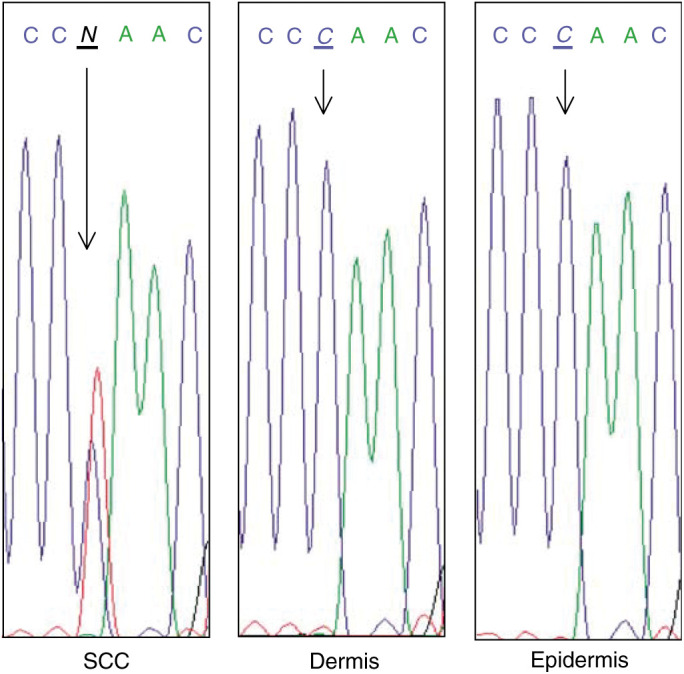
Somatic heteroplasmic mutation in skin mtDNA from a 77-y-old SCC patient. A heteroplasmic C→T mutation at np 438 was identified in the tumour only. The matched histologically normal perilesional epidermis and dermis show only the wild-type sequence.

Unlike the homoplasmic single base changes, the single heteroplasmic base substitution was detected in the tumour only (i.e. SCC from a 77-y-old patient). This is particularly interesting, as it is strongly suggestive of a somatic mutation. It has been previously shown that approximate levels of heteroplasmy can be determined from the sequence electropherogram (Taylor *et al*, 2001). Using this methodology, an approximate value of 70% mutant mtDNA in the SCC sample was estimated. The heteroplasmic change is a C to T transition at np 438 in the noncoding D-loop (Figure 3

), and furthermore it has not been previously reported (MITOMAP). Similarly, 13 of the 81 homoplasmic base changes have not been previously reported ( Table 1A). Three of these homoplasmic changes were located in the noncoding D-loop and 10 in the coding region. Two of these 10 single base transitions occur in the same BCC patient and alter amino acids within the ND5 subunit of the complex I. The A to G transition at np 13528 changes a threonine to an alanine, whereas the C to T transition at np 13565 alters a serine to phenylalanine.

Five of the 68 previously reported base changes (MITOMAP) alter an amino acid in the ND5 (four out of five changes) and ND1 (one out of five changes) genes ( Table 1B). The T to C transition at np 4216 in the ND1 gene was in a BCC (89 y) as well as in an SCC (76 y) patient. Two of the four base transitions within the ND5 gene at np 13105 and 13708 ( Table 1B) are both present in the same BCC patient (76 y). The two remaining base transitions, at np 13117 and np 13934, were observed in a BCC patient (88 y) and an SCC patient (70 y), respectively. Details of the 63 reported base changes that do not alter an amino acid are summarised in Table 1C. Further analysis of these and the other homoplasmic base changes reported in Table 1 showed a lack of correlation with age or category of NMSC (results not shown).

## DISCUSSION

For the first time in the skin cancer literature, we have presented a detailed study of the distribution of multiple forms of mtDNA damage in NMSC.

### The spectrum of large-scale mtDNA deletions

This has been rarely investigated fully in previous tumour studies and certainly not in skin (Penta *et al*, 2001). A total of 20 different deletion species were observed in the major deletion arc and four species were observed in the minor deletion arc. This contrasts markedly with a single study in colorectal carcinomas, which reported an absence of large-scale deletions using a similar long PCR strategy (Penta *et al*, 2001). Although the absolute sample numbers are small, our data clearly show that more deletions occur in the skin from BCC rather than SCC patients. This may be explained through different selection pressures exerted on the deletions. For example, part of the process by which precursor lesions progress to SCCs may represent a mtDNA mutation bottleneck (reviewed in Chinnery *et al*, 2000) where deleterious mutations are selected against. Within each patient, the lowest number of differently sized deletions was observed in the epidermis, which agrees with our previous study that compared dermis with epidermis from normal skin (Ray *et al*, 2000). Interestingly, in contrast to the distribution of the homoplasmic base substitutions described in our study, the observed pattern of deletions in the tumour was often very different compared to that observed in the matched perilesional skin. This may be suggestive of different mutation and segregation events thereby giving rise to different deletion spectra between the tumour and perilesional skin. Interestingly, one of these deletions, derived from the minor deletion arc and approximately 4 kb in size, was present in every skin sample that harboured a deletion. The identity of this deletion may be the 3895 bp species that has been reported in the minor deletion arc spanning nucleotides 547–5443 (MITOMAP), and is associated with Kearns Sayre Syndrome and Chronic Progressive External Ophthalmoplegia.

### The 4977 bp common mtDNA deletion and tandem duplications

The common deletion has been reported in 52% of 32 gastric carcinomas, which is in marked contrast to the low incidence of large-scale deletions in other tumours (Penta *et al*, 2001). The authors of the gastric study have explained their observations by the use of a more sensitive PCR technique, which detects low levels of the common deletion (Maximo *et al*, 2001). It is well known, however, that studies of low levels (i.e. typically <0.2%) of mtDNA deletions can be confounded by the effects of ageing (Birch-Machin *et al*, 1998; Nagley and Wei, 1998; Chinnery *et al*, 1999). This scenario was avoided in our study by simply presenting only high levels (>2%) of the common deletion. We have previously used this approach to show that this threshold value does not reflect chronological ageing (Birch-Machin *et al*, 1998).

The present study is the first quantitative investigation of the incidence of the common deletion in tumours. Higher deletion levels in tumour were observed for three of the four patients who harboured high levels of the deletion in both tumour and perilesional skin. The observed pattern of the common deletion contrasts once again with the distribution of the homoplasmic base substitutions between tumour and perilesional skin. Unlike a previous gastric study (Maximo *et al*, 2001), we could find no evidence of an inverse relation between the incidence of the common deletion and single base changes (results not shown).

There has been a suggested link between the presence of the common deletion and tandem duplications in the D-loop (Brockington *et al*, 1993). Two of the seven NMSC patients, who harboured high levels of the common deletion, also contained either a 200 or 260 bp tandem duplication. To our knowledge, this is the first report of tandem duplications of this type in tumours. It is known that the tandem duplication encompasses several regulatory regions, including the light- and heavy-strand promoters, binding sites for mitochondrial transcription factor 1 and most of the conserved sequence box II. The significance of these findings is unclear until more work is performed within the field of tandem duplications and skin (Krishnan and Birch-Machin, 2002).

### MtDNA base changes

A total of 81 homoplasmic and one heteroplasmic single base changes were identified in the skin from the NMSC patients. Perhaps, the most interesting of these is the previously unreported heteroplasmic transition at np 438 that was observed in an SCC but was absent from the histologically normal perilesional tissue. This is highly suggestive of a somatic mutation of the kind that has been previously reported in other tumour types (Penta *et al*, 2001). The somatic mutation occurs within a region of the D-loop, which encompasses the origin of H-strand replication, the light-strand promoter and a binding site for mitochondrial transcription factor 1. This suggests that the base alteration at np 438 may have a profoundly deleterious effect on mitochondrial function. The mechanism by which the heteroplasmic state of the np 438 has reached a value of 70% is unknown. It has been shown that a selective cellular growth advantage can be conferred by mutations in the mitochondrial genome that can result not only in a high level of heteroplasmy but also homoplasmy for the mutation (Polyak *et al*, 1998). A contrasting model has recently been put forward to explain this phenomenon (Coller *et al*, 2001). The authors suggest that high levels of a heteroplasmic, and indeed a homoplasmic, mitochondrial mutation in a tumour can result from random segregation of mutant genomes in the many cell generations that occur during tumour development.

Each of the 81 identified homoplasmic substitutions were observed both in the tumour and perilesional skin. In all, 13 of these 81 changes are unreported in the MITOMAP database. More than half (44 out of 81) of the single base changes was observed in the noncoding D-loop region. There are several possible explanations for the presence of these 81 homoplasmic changes in both tumour and perilesional skin. The first explanation may simply be benign polymorphisms completely unrelated to the tumour formation. In support of this, it has been suggested that sequencing the mitochondrial genomes of random Europeans would reveal approximately 10–12 single base changes between individuals (personal communication, Dr Neil Howell, MitoKor, San Diego, USA). An alternative explanation may be the fact that all the skin samples used in our study are taken from constant sun-exposed body sites and as such will have potentially received the same mutagenic dose of UVR. This could be resolved through sequencing mtDNA isolated from sun-protected sites, but current ethical permission for the project does not allow the recall of the skin cancer patients for this intended purpose.

We have identified seven base changes that introduce an amino-acid change within the genes for ND5 (*n*=6) and ND1 (*n*=1). Two of these at np 4216 (ND1) and 13708 (ND5) have been correlated with Lebers Hereditary Optic Neuropathy (MitoAnalyzer; MITOMAP, National Institute of Standards and Technology, Gaithersburg, MD, USA). In addition, two further base substitutions at np 13528 and 13565 were previously unreported, and both of these alter an amino acid with a polar side chain to one with a nonpolar side chain. The functional significance, if any, of these alterations is at present unclear, particularly as these amino acid changes are present both in the tumour and the perilesional skin. However, like all the other previous mtDNA and cancer studies in the literature, the data are derived from automated DNA sequencing that will reliably detect mutant loads only as low as 30% (Taylor *et al*, 2001). Therefore, below the 30% threshold value there might be differences in mutagenic load between tumour and perilesional skin. This is important for two reasons. First, there is a threshold effect that describes the critical ratio between wild-type and mutant mtDNA, which must be exceeded before phenotypic expression of the mutant becomes apparent. For protein coding mtDNA genes, such as those described in our study, the threshold value is around 70% and above (Chomyn *et al*, 1992). Secondly, given the large number of cell divisions necessary for tumour development, estimated at 600 by Coller *et al*, (2001), an alteration in mitochondrial function that is exceedingly subtle in terms of its biochemical or physiological manifestation may be all that is necessary to alter significantly the probability of tumour formation (Augenlicht and Heerdt, 2001).

An increased incidence of C insertions/deletions, within a homopolymeric C-stretch between np 303 and 315 of the D-loop (i.e. D310 tract), has been recently reported in several cancers (Parrella *et al*, 2001; Sanchez-Cespedes *et al*, 2001). These studies have proposed that instability in the D310 tract may be a useful diagnostic marker for cancer development. In skin however, we have observed that instability in the D310 tract is present not only in the tumour but also in the histologically normal perilesional skin. Both skin sites receiving the same mutagenic dose of UVR may explain this phenomenon. However, D310 instability is observed regularly in mitochondrial genomes from noncancerous skeletal muscle and brain (personal communication, Dr R Taylor, Mitochondrial Gene Therapy Group, Newcastle, UK). Given these observations, more work is therefore needed before D310 instability can be claimed to be a reliable and universal diagnostic marker for cancer.

In conclusion, we provide the first detailed study of the distribution of multiple forms of mtDNA damage in NMSC and histologically normal perilesional tissue. We present the first entire spectrum of deletions found between different types of skin tumours and perilesional skin. In addition, we provide the first quantitative data for the incidence of the common deletion as well as the first report of specific tandem duplications in tumours from any tissue. This work shows that there are differences in the distribution of deletions between the tumour and the histologically normal perilesional skin. DNA sequencing of four mutation ‘hotspot’ regions of the mitochondrial genome identified a previously unreported somatic heteroplasmic mutation in an SCC patient. In addition, 81 previously unreported and reported homoplasmic single base changes were identified in the other NMSC patients. Unlike the distribution of deletions and the heteroplasmic mutation, these homoplasmic mutations were present in both tumour and perilesional skin. This suggests that the traditional use of histologically normal perilesional skin in NMSC studies may have several limitations. This is important when one considers that the majority of studies involving nuclear DNA damage and skin cancer/skin disease often use perilesional skin as a control tissue. Currently, it is unclear whether mtDNA damage has a direct link to skin cancer or it may simply reflect an underlying nuclear DNA instability.
